# Does the polyubiquitination pathway operate inside intact chloroplasts to remove proteins?

**DOI:** 10.1093/plcell/koae104

**Published:** 2024-04-29

**Authors:** Klaas J van Wijk, Zach Adam

**Affiliations:** Section of Plant Biology, School of Integrative Plant Sciences (SIPS), Cornell University, Ithaca, NY 14853, USA; Faculty of Agriculture, Institute of Plant Sciences, The Hebrew University of Jerusalem, Rehovot 76100, Israel

Dear Editor,

The Ubiquitin Proteasome System (UPS), discovered by Hershko and Ciechanover ∼45 yr ago (Ciehanover et al. 1978), has long been recognized as the major protein degradation pathway within all eukaryotic cells. As such, it is involved in almost all aspects of life in eukaryotic organisms, including plants (reviewed in, e.g. Hershko and Ciechanover 1998; Moon et al. 2004; Smalle and Vierstra 2004; Vierstra 2009; Komander and Rape 2012; Sadanandom et al. 2012; Cappadocia and Lima 2018; Miricescu et al. 2018). In a nutshell, the UPS consists of the highly conserved 76 amino acid ubiquitin protein (Ub) that can be adenylated by an E1 enzyme, and then, with or without an E2 Ub-conjugating enzyme, and an obligatory E3 Ub ligase, the activated Ub is attached to a substrate protein. This sequence of reactions repeats itself to yield polyubiquitinated proteins, that are, in a process requiring additional ATP, recognized, unfolded, and degraded by the 26S proteasome. The UPS operates in the cytosol and the nucleus, but it can also degrade endoplasmic reticulum (ER) resident proteins that are extracted and translocated back for degradation in the cytosol in a process known as ER-associated protein degradation (Strasser 2018; Wu and Rapoport 2018; Manghwar and Li 2022). Here, misfolded luminal or integral membrane proteins move across the ER membrane through a channel made of specific Ub ligase molecules, and following polyubiquitination on the cytosolic side of the membrane, they are extracted by the cytosolic AAA + ATPase CDC48 and delivered for degradation by the proteasome. The activity and targeting of CDC48 are controlled by adaptor proteins such as PUX proteins (Zhang et al. 2021). In recent years, a chloroplast homologous system, designated chloroplast-associated protein degradation (CHLORAD), was discovered (Ling et al. 2019; Sun and Jarvis 2023). This system, located at the chloroplast outer envelope membrane, consists of 3 major components: (i) SP1, an E3 Ub ligase that can ubiquitinate proteins belonging to the TOC complex (mediating the import of proteins into chloroplasts), (ii) a β-barrel channel composed of SP2 proteins, and (iii) a cytosolic CDC48 unfoldase complex capable of extracting the polyubiquitinated proteins from the outer envelope membrane and directing them for degradation by the cytosolic 26S proteasome (Ling et al. 2019). CDC48 also serves nonchloroplast targets, and its homologs in nonplant eukaryotes (also named VCP or p97) have a similar molecular function as a major cytosolic protein unfolding machine (Meyer and van den Boom 2023). This CHLORAD pathway is important for chloroplast differentiation, conversion of chloroplasts to gerontoplasts during senescence or to chromoplasts during tomato fruit ripening (Ling et al. 2021), and in response to different stresses (Sun and Jarvis 2023). The UPS is also involved in the degradation of nuclear-encoded chloroplast precursor proteins, en route to the chloroplast. If they fail to import into chloroplasts, such precursor proteins are polyubiquitinated in the cytosol by the E3 ligase CHIP and degraded by the 26S proteasome (Shen et al. 2007a, 2007b).

Recently, the role of CHLORAD was proposed to extend to the degradation of proteins residing within intact chloroplasts as well (Li et al. 2022; Sun et al. 2022). In Li et al. (2022), it is claimed that polyubiquitinated proteins are found inside intact chloroplasts, including proteins encoded by the plastid genome, such as RbcL and AtpB. Moreover, their level is modulated by CDC48 through their degradation by the proteasome (Li et al. 2022). Yet, the authors acknowledge that this polyubiquitination is unlikely to happen inside intact chloroplasts because there are no known E1–E2–E3 enzymes in the chloroplast, nor a protein export channel in the inner envelope membrane, thereby undermining their own model. An even stronger claim is made in Sun et al. (2022), concluding that a wide array of photosynthetic proteins (stroma and thylakoid localized), including many chloroplast-encoded proteins, are polyubiquitinated inside intact chloroplasts (even on lysine residues exposed to the thylakoid luminal space) and subsequently degraded in the cytosol by the proteasome (Sun et al. 2022). As the long-standing dogma in the field has been that the UPS does not operate inside intact chloroplasts (Beers et al. 1992; Ramos et al. 1995; Adam 1996), these surprising publications (Li et al. 2022; Sun et al. 2022) prompted us to reevaluate this dogma and its history, and the newly published experimental evidence supporting its abolishment. If the role of CHLORAD is to be extended to the degradation of proteins residing inside intact chloroplasts, 3 premises are expected: (i) free Ub should be found in the chloroplast stroma; (ii) E1–E2–E3 enzymes, necessary for protein ubiquitination, should be located inside chloroplasts; and (iii) a protein export pathway, with specificity to polyubiquitinated proteins, should locate and operate in the inner envelope membrane, before such potential substrates can be engaged with existing components of the CHLORAD system located in the outer envelope membrane and cytosol. If the proposed results and conclusion are true, it would signal a major change in the field of proteolysis/proteostasis, breaking the long-standing dogma that there are no polyubiquitination mechanisms within intact chloroplasts.

In the following paragraphs, we will briefly review the older attempts to identify the components of the UPS in intact chloroplasts and the results suggesting that ubiquitination does not occur inside chloroplasts. We will then briefly evaluate the experimental data of these 2 recent studies (Li et al. 2022; Sun et al. 2022) and discuss whether the suggestion that proteins can be ubiquitinated inside intact chloroplasts and retrieved by CHLORAD for degradation by the 26S proteasome in the cytosol is indeed justified.

## Early work

Published attempts to identify Ub or ubiquitinated proteins in chloroplasts already appeared in the 1990s, soon after the discovery of the UPS. Immunoelectron microscopy of *Chlamydomonas* cells or tobacco leaf sections with antibodies against Ub revealed labeling in different cell compartments, including chloroplasts (Gaspar et al. 1990; Wettern et al. 1990). However, immunogold signals were distributed almost equally in all cellular compartments, or particles were especially difficult to recognize against the dark-stained and granular backgrounds. Moreover, an immunoblot analysis was performed on whole cell extracts rather than on isolated organelles, making the claim for having free Ub or ubiquitinated chloroplast proteins inside chloroplasts highly questionable. In another study, Ub-conjugating activity was claimed in chloroplasts isolated from oat leaves (Veierskov and Ferguson 1991). Here again, no attempt was made to distinguish between conjugating activities inside chloroplasts and those that might have been associated with the outer surface of the organelle, either under physiological conditions or due to cytosolic contamination during the organelle isolation procedure. Following isolation of a tomato cDNA clone encoding a Ub protein, immunological studies of isolated and fractionated chloroplasts were performed (Hoffman et al. 1991). Free Ub could not be detected in protease-treated isolated chloroplasts. However, higher-molecular-weight protein bands in isolated chloroplasts were observed by immunoblotting and interpreted as an indication for the possibility of import of ubiquitinated proteins into chloroplasts. Perhaps the most comprehensive attempt to localize ubiquitinated proteins in different cellular locations at that time was made by Beers et al. (1992). In this study, gel patterns of ubiquitinated proteins were compared between purified subcellular fractions and unfractionated Arabidopsis extracts. Although purified nuclei and vacuoles contained significant amounts of ubiquitinated proteins, cell wall proteins and chloroplast stromal proteins did not have signals above cytosolic contamination background. Similarly, an even more extensive study with intact chloroplasts, isolated from *Lemna minor* and spinach by Percoll density gradient centrifugation, detected neither free ubiquitin nor ubiquitin–protein conjugates (Ramos et al. 1995). This study used both immunoblotting and ^125^I-labeled ubiquitin conjugation assays. Furthermore, treatment of the mechanically isolated chloroplasts with thermolysin suggested that the presence of ubiquitin–protein conjugates in some chloroplast preparations is due to cytosolic (or outer envelope protein) contamination. Importantly, this study verified and confirmed the integrity of the isolated chloroplasts (using the classic spectrophotometric ferricyanide reduction assay) as well as the specificity of the antiubiquitin antibody response (using, e.g. preimmune sera and positive controls). This study also commented on previous reports on ubiquitination within chloroplasts and identified pitfalls in them. Thus, the accepted view since that time has been that chloroplasts contain neither free Ub nor the enzymatic machinery necessary for its conjugation to potential substrate proteins (Beers et al. 1992; Ramos et al. 1995; Adam 1996).

## Are components of UPS and polyubiquitinated proteins found inside chloroplasts?

In Arabidopsis, as well as in other species, Ub is encoded by multi-gene families, giving rise to the highly conserved 76 amino acid Ub protein. Given the very high conservation of Ub proteins, widely available antibodies have been traditionally used to detect free Ub as single bands at ∼8.5 kDa or polyubiquitinated proteins that usually appear as a wide array of bands and smears in immunoblot analyses (Wettern et al. 1990; Beers et al. 1992; Ramos et al. 1995). Li et al. (2022) used this approach to try and detect ubiquitinated proteins in total leaf extracts and isolated intact chloroplasts (Li et al. 2022). Using an antibody against Ub11 (Li et al. 2022), distinct bands were detected in whole chloroplasts and in fractionated chloroplasts isolated from different genetic backgrounds, or in samples from seedlings treated with an inhibitor of the 26S proteasome (see Fig. 1 in Li et al. 2022). Unfortunately, in all these experiments, no attempt was made to remove ubiquitinated proteins from the outer surface of the isolated chloroplasts. Thus, the observed signals could be of outer envelope membrane proteins, either integral to the membrane or peripherally attached to it, a possibility that is supported by the detection of cytosolic CDC48 in these chloroplast samples (Fig. 1A in Li et al. 2022). Moreover, no attempt was made to unravel the identity of the purportedly ubiquitinated protein bands by an independent method such as mass spectrometry (MS). Sun et al. (2022) circumvented the possibility of having the ubiquitination signals coming from outer envelope proteins by treating the isolated chloroplasts with the protease thermolysin, which removes peripheral proteins or cytosol-exposed regions of integral membrane proteins from the outer surface of the organelle. Unfortunately, they immunoblotted with an anti-myc antibody (rather than anti-Ub) following the expression of a Myc-tagged Ub (6Myc-Ub) under the control of the 35S promoter in transgenic plants. There is no indication in the article for the inclusion of a chloroplast-targeting sequence in this chimeric protein. Thus, it is not clear whether or how this 6Myc-Ub gets into chloroplasts. The robust expression of the chimeric construct in the cytosol could potentially result in ubiquitination of chloroplast precursor proteins prior to their import into the organelle. It should also be noted that no free Ub was detected in isolated chloroplasts in this study.

We are not aware of any attempt to detect Ub-conjugating and Ub-ligating enzymatic activities in isolated chloroplast fractions, nor of any reports of identification of E1, E2, or E3 proteins by immunoblotting or proteomics, ever since the older work mentioned above was published (Veierskov and Ferguson 1991). An inspection of public proteomics databases such as SUBA and PPDB does not suggest any of these proteins to be located in chloroplasts. Previously, we used an mRNA-based co-expression network analysis to determine whether any E3 ligases are possibly involved with chloroplast proteostasis (Majsec et al. 2017). We found 5 candidate E3 ligases (SC-F box AT5G63780 and AT5G58580; RING-type AT1G57790, AT2G04230, and AT4G07400) that showed co-expression with chloroplast proteins (known chloroplast proteins often co-express with other chloroplast proteins). However, based on the literature, the lack of chloroplast signal peptides, and information from the latest proteomics resource Arabidopsis PeptideAtlas release 2023-10 (https://peptideatlas.org/), these E3 ligases are unlikely to be localized within plastids, even if their co-expression with known plastid proteins suggests that they are somehow involved with plastid protein homeostasis.

Taken together, the specific and shared limitations of the 2 new studies described here (Li et al. 2022; Sun et al. 2022), together with the lack of any indication for the presence of free Ub and the obligatory E1–E2–E3 enzymatic system inside chloroplasts, cast a shadow on the suggestions that ubiquitination of chloroplast proteins occurs within the intact organelle.

## MS-based evidence of polyubiquitinated proteins inside chloroplasts

The conclusion in Sun et al. (2022) that intra-chloroplast proteins in Arabidopsis can be polyubiquitinated and then extracted into the cytosol for subsequent degradation by the proteasome relies strongly on several sets of tandem mass spectrometry (MSMS) data. As these comprise the first report on ubiquitinomics of chloroplast proteins, it prompted a reanalysis of these raw MSMS data using both open and closed sequence database searches (van Wijk et al. 2023). We encountered not only many issues with the reported results but also discrepancies between stated methods (e.g. use of the alkylating agent iodoacetamide [IAA]) and observed mass modifications (van Wijk et al. 2023). The Reply Letter from Jarvis et al. now comments on the issue of their use of alkylating agents and it is important to explain this issue to nonexperts. Identification of ubiquitinated proteins (using standard proteomics workflows with affinity enrichment of ubiquitinated proteins followed by digestion with trypsin and nanoLC-MSMS) typically relies on the detection of a mass modification of +114.0429 Da on the side chains of lysine residues. This delta mass results from the attachment of 2 glycine residues from the C-terminal portion of Ub (K-ε-GG; the “diglycine footprint”). Unfortunately, covalent attachment of 2 acetamide molecules to lysine results in an identical mass modification (Nielsen et al. 2008). Such lysine modifications can occur during the alkylation reaction to block the sulfhydryl of cysteine residues (a standard procedure in proteomics), particularly when using IAA (Nielsen et al. 2008; Hains and Robinson 2017). The frequency of off-target alkylation can be reduced by using lower IAA concentrations at lower temperatures, or by using chloroacetamide. We refer to van Wijk et al. (2023) for a more in-depth analysis and discussion on this issue.

## Protein export pathways from chloroplasts

From all we know about the well-studied TOC-TIC pathway, which is responsible for the import of most chloroplast proteins into the organelle, translocation across the 2 membranes comprising the chloroplast envelope is unidirectional, from the cytosol into the chloroplast (Kim et al. 2023; Sun and Jarvis 2023). Currently, there is no known pathway for the export of (un)folded proteins from the chloroplast interior to the cytosol. However, multiple vesicle–mediated pathways are known to deliver chloroplast contents, or even whole chloroplasts, through the cytosol to the vacuole for degradation (Izumi and Nakamura 2018; Otegui 2018; Zhuang and Jiang 2019; Fu et al. 2022). These are divided between autophagosome-mediated pathway (rubisco containing [RBC] bodies, ATG8-INTERACTING PROTEIN1 plastid [ATI-PS] bodies, starch small starch granule-like [SSGL] bodies) and autophagosome-independent pathway (senescence-associated vacuoles [SAVs] and chloroplast-vesiculation-containing vesicles [CCVs]). The degradation of such chloroplasts and chloroplast proteins likely takes place within vacuoles, and it is not clear whether the cargo of any of these vesicles may be released in the cytosol and eventually polyubiquitinated and degraded by the proteasome. Although mechanistic details of most of these vesicle-mediated pathways are still missing, they are particularly important in response to stresses such as nutrient stress (e.g. nitrogen limitation, loss of photosynthesis), light stress, and exposure to UV radiation and/or senescence. In addition to these vesicle pathways, the cytosolic PUB4 E3 ligase is involved in the removal of damaged chloroplasts upon increased accumulation of reactive oxygen species (ROS). Under these circumstances, the outer surface of damaged chloroplasts is ubiquitinated by PUB4 (Woodson et al. 2015). However, it was not determined how these ubiquitinated chloroplasts are degraded, and whether this degradation involved autophagy. A recent paper from Izumi and colleagues suggested that such PUB4-associated ubiquitination is dispensable for the induction of autophagy and suggests parallel functions of PUB4 and chlorophagy (Kikuchi et al. 2020; Nakamura and Izumi 2021). In chloroplast mutants affected in chloroplast proteostasis (*gun1* and *ftsh5-3*), PUB4 was shown to contribute to the degradation of damaged chloroplasts, but the mechanism was not determined (Jeran et al. 2021).

## Loss of chloroplast integrity can result in chloroplast protein ubiquitination

Previous studies have demonstrated that high levels of ROS, produced due to excess light treatment or due to ROS generated from light-absorbing chlorophyll intermediates such as in the *flu* mutant, result in damage, swelling, loss of chloroplast integrity, and possible rapid release of chloroplast proteins into the cytosol, as demonstrated by confocal microscopy of plants expressing chloroplast-targeted Green Fluorescent Protein (GFP) in the *flu* mutant (Danon et al. 2005; Kim et al. 2012). Similarly, a recent study suggested that short-term exposure to extreme light stress (from 40 to 1,500 *μ*mol m^−2^  s^−1^) combined with reduced temperature (from 22 to 12 °C) resulted in the loss of chloroplast envelope integrity (Lee et al. 2023). It was postulated that this allowed unidentified cytosolic E3 ligase(s), but not envelope E3 ligase SP1 or PUB4 ligase, to reach the chloroplast interior and ubiquitinate thylakoid and stroma proteins. Under these circumstances, the autophagy receptor NBR1 was found not only on the outside of chloroplasts but also inside the damaged chloroplasts. It was postulated that these polyubiquitinated chloroplast proteins are recognized by NBR1 for micro-autophagic clearance independent of ATG7, and therefore, of ATG8 lipidation (Lee et al. 2023). Hence, under conditions where chloroplasts are severely damaged and with “broken” envelope membranes, it is possible that stromal chloroplast proteins can be ubiquitinated.

## Are there lessons from mitochondrial ubiquitination-dependent degradation pathways?

Beyond the mitochondrial outer membrane, proteasome-mediated degradation has been suggested to play a role in the protein quality control of the mitochondrial intermembrane space, inner membrane, and matrix compartments, in particular in yeast. In yeast, dysfunctional intermembrane space proteins that are insufficiently oxidized are retro-translocated from the intermembrane space compartment to the outer membrane, where they are ubiquitinated and degraded by the proteasome (Uoselis et al. 2023). How intermembrane space, inner membrane and matrix-localized proteins undergo selective retro-translocation to the surface of mitochondria remains to be determined, including the question of whether they transit through the mitochondrial translocases of the outer and inner membranes. It is important to point out that yeast mitochondria lack a Clp protease system, whereas plant and human mitochondria have a relatively abundant matrix-localized ClpP protease and several ClpX chaperones involved in the selection and delivery of substrates to the matrix protease. In human mitochondria, the Clp system has been shown to be important for health, and loss of Clp protease activity is the cause of several inheritable diseases (Mabanglo et al. 2022). Hence, it is quite possible that mitochondria in species that lack a Clp system (but still contain the Lon/Pim and FtsH/mAAA-iAAA proteases), such as yeast, might have evolved retro-translocation (from intra-mitochondrial spaces to the cytosol) to cope with the lack of a major matrix-located proteolysis system. Various types of mitophagy (ATG-dependent and ATG-independent) complement intra-mitochondrial protein turnover pathways, similar to observations in chloroplasts. A few reports claim that yeast mitochondrial matrix proteins are polyubiquitinated inside mitochondria, but this is certainly not a widely accepted view and is essentially ignored in recent expert reviews (Rodl and Herrmann 2023; Uoselis et al. 2023).

## Take-home message

Neither older publications nor the recent papers provide evidence for the presence of free Ub and E1–E2–E3 proteins inside intact chloroplasts. There is no robust evidence for the presence of polyubiquitinated chloroplast proteins within the intact organelle (van Wijk et al. 2023). However, chloroplast proteins can be ubiquitinated (i) in the cytosol en route to the chloroplast, (ii) when chloroplasts lose their membrane integrity (e.g. by ROS damage), or (iii) once chloroplast protein content is removed from chloroplasts through various vesicle transport mechanisms for degradation by extra-plastid proteolytic machineries such as the proteasome or vacuolar proteases. An extensive protease network (protease web) operating inside chloroplast subcompartments further supports chloroplast protein homeostasis and complements extra-plastid degradation pathways, involving the UPS system and micro- and macro-autophagy (van Wijk 2015; Majsec et al. 2017; Nishimura et al. 2017; Fu et al. 2022; Gao et al. 2023).

## Dive Curated Terms

The following phenotypic, genotypic, and functional terms are of significance to the work described in this paper:

CDC48 Gramene: AT3G09840
CDC48 Araport: AT3G09840

ATP CHEBI: CHEBI:15422

FLU Gramene: AT3G14110
FLU Araport: AT3G14110

PUB4 Gramene: AT2G23140
PUB4 Araport: AT2G23140

GUN1 Gramene: AT2G31400
GUN1 Araport: AT2G31400
